# Massive Right Atrial Thrombosis: Are You Brave Enough to Start Anticoagulation? A Case Report

**DOI:** 10.3389/fcvm.2021.688351

**Published:** 2021-07-30

**Authors:** Marco Bergonti, Ciro Ascione, Piergiuseppe Agostoni, Roberto Castelli, Carlo Vignati

**Affiliations:** ^1^Cardiovascular Disease Fellowship Program, University of Milan, Milan, Italy; ^2^Monzino Cardiology Center, Istituto di Ricovero e Cura a Carattere Scientifico, Milan, Italy; ^3^Department of Clinical Sciences and Community Health, Cardiovascular Section, University of Milan, Milan, Italy; ^4^Department of Biomedical and Clinical Sciences, Luigi Sacco Hospital, University of Milan, Milan, Italy

**Keywords:** right atrial thrombosis, pulmonary thromboembolism, thrombus, cardio-oncology, coagulation, right atrium mass

## Abstract

Chronic myelomonocytic leukemia (CMML) is a clonal hematopoietic stem cell disorder with overlapping myelodysplastic and myeloproliferative features. The disease is generally characterized by blood monocytosis, bone marrow dysplasia, cytopenia, and hepatosplenomegaly. While malignant blood diseases are frequently associated with a high risk of thromboembolism, CMML is often accompanied by immune-mediated hemorrhagic diathesis. Indeed, very few reports in literature report thrombotic complications of CMML patients. We will briefly present here the case of a patient with CMML who developed a massive right atrial thrombus. We aim to highlight the non-negligible thrombotic burden of the disease, and we will get through the differential diagnosis of right atrial masses and the management of right atrial thrombi, which are a rare and poorly known entity.

## Introduction

Chronic myelomonocytic leukemia (CMML) is a clonal hematopoietic stem cell disorder with myelodysplastic and myeloproliferative overlap features. The disease is generally characterized by blood monocytosis, bone marrow dysplasia, cytopenias, and hepato-splenomegaly (1–4).

Of note, CMML is often accompanied by immune-mediated hemorrhagic diathesis while thrombotic manifestations are rare (1, 4), especially involving massive spontaneous thrombosis in the right atrium. Indeed, this condition has only been described once in the literature (5). We report below the second of such cases.

## Case Description

A 78-year-old male was admitted to our hospital for increasing fatigue, multiple episodes of syncope, and paroxysmal episodes of dyspnoea. He was known for CMML diagnosed in 2016 and currently treated with supportive care. He had markedly enlarged spleen (20 cm of diameter) and severely reduced hemoglobin and platelet level. Chemotherapy had not been deemed suitable due to comorbidity.

His past medical history also comprised heart failure with mid-range ejection fraction (HFmEF) secondary to anterior myocardial infarction. He had previously underwent double-chamber pacemaker implantation for critical bradycardia in 2010. He had severe aortic stenosis and stage III chronic kidney disease (CKD). He did not have persistent or paroxysmal atrial fibrillation, a history of thromboembolism, or a family history of coagulopathy. He was on therapy with Clopidogrel 75 mg q.d., Levo-tiroxin 150 mcg q.d., tamsulosin 0.4 mg q.d., pantoprazole 40 mg q.d., furosemide 25 mg q.d., spironolactone 25 mg q.d., sacubitril/valsartan 24/26 mg b.i.d., silodosin 8 mg q.d., allopurinol 150 mg q.d., calcitriol 0.5 mcg q.d., and erythropoietin 4,000 IU twice per week.

Upon arrival in the emergency department (ED), he appeared pale and diaphoretic. His blood pressure was 100/60 mmHg, heart rate was 80 bpm, and oxygen saturation was 96%. His physical examination was remarkable only for a harsh systolic murmur radiated to both carotids. His lungs were clear and no lower limb edema was evident. His labs upon admission in the ED were as follows: white blood cell count 11,300/μl with 32% monocyte, platelets 47,000/μl, hemoglobin 9.3 g/dl, PT 16.8 s, INR 1.44, aPTT 37 s, fibrinogen 123 mg/dl [150–450], antithrombin III 58.0% [80–120], D-dimer 1,732 ng/ml, and creatinine 3.08 mg/dl (eGFR 18 ml/min).

**Figure 1:**
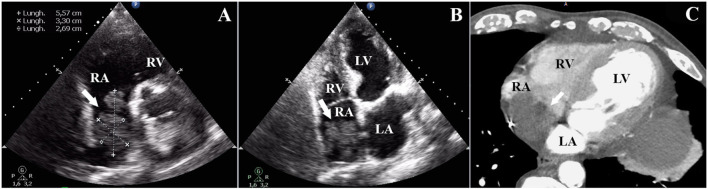
**(A,B)** Modified short-axis view and four-chamber view showing irregular thrombotic mass pointing toward the tricuspid valve, into the right ventricle. **(C)** Venous phase, contrast CT scan with evidence of intraluminal hypo-dense filling defect, with irregular profile, nearly occluding an enlarged right atrium.

In the ED, he underwent transthoracic echocardiography (TTE) showing a dilated right atrium containing a 5.57 × 3.30 × 2.69 cm pedunculated mass, with irregular heterogeneous surface, attached to the superior atrial wall, partially crossing and nearly occluding the tricuspid valve (Figures 1A,B). The superior and inferior vena cava were not involved. Contrast CT described an intraluminal hypodense filling defect, with irregular profile, sparing the superior and inferior vena cava and being apparently attached to the atrial catheter of the pacemaker and the atrial portion of the ventricular catheter (Figure 1C). Mass density was compatible with thrombus apposition. Venous color Doppler ultrasound of the lower extremity was negative.

**Figure 2:**
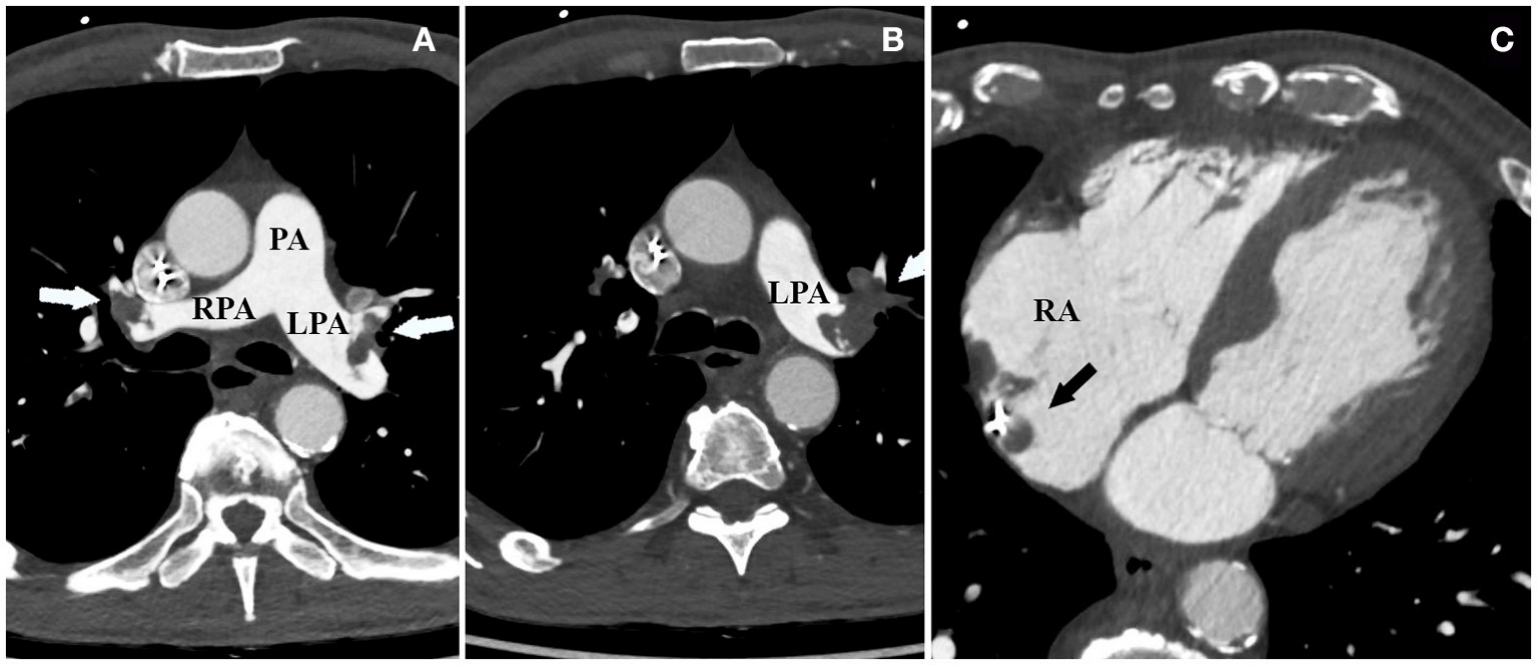
**(A,B)** Bilateral thrombotic apposition nearly occluding the pulmonary arteries and causing bilateral massive pulmonary embolism. **(C)** Nearly complete detachment of the thrombus from the right atrium, which is now only in part visible in proximity to the atrial catheter.

Based on echocardiography and tomography characteristics, the hypothesis of spontaneous thrombus in the right atrium, although extremely rare, was favored as the most probable diagnosis for this giant atrial mass. After Heart Team discussion, all open heart surgical options were excluded due to the prohibitive risk associated with his comorbidities. In light of stable hemodynamic conditions, fibrinolytic therapy was ruled out as well. Subcutaneous anticoagulation with unfractionated heparin (UH) was thus started. Two days later, respiratory insufficiency and metabolic acidosis suddenly developed. Pulmonary embolism secondary to the detachment of thrombi debris from the right atrium was quickly suspected. Emergency CT angiography showed a complete detachment of the atrial thrombus into the pulmonary arteries (Figures 2A,B). The same exam showed the presence of small residual thrombotic apposition across the atrial catheter (Figure 2C). The patient was slowly stabilized. He remained in the intensive care unit (ICU) for 25 days and was discharged home after 38 days of hospitalization on oral anticoagulation with warfarin plus clopidogrel 75 mg.

## Timeline

**Table:** 

2016	The patient was diagnosed with chronic myelomonocytic leukemia
July 2019 (Day of the admission)	The patient was admitted to the hospital for increasing fatigue and syncopal episodes
Day of the admission: 11.00 a.m.	Diagnosis of right atrial mass at transthoracic echocardiography
Day of the admission: 2.00 p.m.	Contrast CT confirmed the presence of right atrial thrombus. Anticoagulation is started.
2 days after the admission	Detachment of thrombi debris from the right atrium caused massive pulmonary embolization
25 days after the admission	The patient was discharged from the ICU
37 days after the admission	The patient was discharged at home.

## Discussion

The case here reported shows an extremely rare complication of CMML, which has only been described once in the medical literature (5).

Oncologic patients are notoriously predisposed to thromboembolic events, and this holds true even for hematologic conditions. Indeed, venous thromboembolism (VTE) is a frequent complication found in ~11% of patients with acute leukemia (3). However CMML confers the patient a significant bleeding risk, compared to a poorly recognized thrombotic risk (1, 4).

Among the different conditions predisposing to bleeding risk, we have to cite the fact that dysplastic macrophages tend to attach factor X leading to an acquired factor X deficiency (1). Autoimmune acquired hemophilia is another rare but remarkable condition, which has been described (6). Indeed case reports of thromboembolic complications in CMML patients are scarcely reported in the literature, especially when dealing with extremely rare sites of thrombus formation, such as the right atrium. Only recently has the pro-coagulant effect of pathologically activated monocytes been demonstrated (7).

Right atrial (RA) masses are a rare entity. The differential diagnosis of RA masses must include three conditions: benign or malignant neoplasm, tricuspid valve vegetation, and thrombus (8).

While on the left side, thrombi are predominantly located into the appendage, the RA presents some peculiar features. Indeed, the right appendage is broad-based and less deep. Because of this, thrombus formation is less common. In addition, on the right side, thrombi usually originate in patients with atrial fibrillation or pro-thrombotic state. Other conditions that predispose to right atrial thrombus include tricuspid prosthesis or stenosis of the tricuspid valve, atrial septal closure devices, and central venous lines. Right-sided pacemaker leads confer a mildly higher risk of spontaneous thrombi, often in association with other risk factors (9). Thromboembolic events are possible adverse effects of erythropoietin administration; however, to our knowledge, it was never associated with atrial thrombosis (10). No other medications taken by the patients are associated with thrombotic risk.

When dealing with RA thrombi, one has to bear in mind that they can be divided into two categories: type A thrombi originate in deep peripheral veins and transit the RA (extremely mobile, worm-shape); type B thrombi, originating into the right atrium, are attached to the atrial wall and immobile. The distinction carries clinical significance as Type A patients are a high-risk group that warrants surgery or fibrinolysis. Type B thrombi are much more benign and vitamin K antagonists seem to be sufficient (9).

Our patient presented with a spontaneous right atrial thrombus, a rare condition that is made nearly unique by the concomitant presence of CMML. Indeed, our patient had no other potential risk factors for developing right atrial thrombus except for pacemakers leads. Genetic tests for hypercoagulable disorders were negative. No tricuspid valve disease or atrial septal defect closure device was present. Thrombophilia screening, including factor V Leiden, MTHFR, and the G20210A mutation of thrombin, was negative.

In light of his history and these findings, we can infer that his lymphoproliferative disease may be the cause of the thrombotic event, and this is the second of such cases ever reported in the medical literature.

In the literature, there are different recommendations for right heart thrombosis treatment: surgical removal, the administration of thrombolytic agents, or anticoagulation therapy with heparin, with similar mortality rates (38, 38, and 30%, respectively); a significantly lower probability of short-term survival is described in untreated patients (19% in patients with pulmonary emboli and 53% in those without pulmonary emboli) (11). Given the high bleeding risk of patients with CMML, anticoagulation seems to be the best alternative.

Another treatment possibility is the AngioVac thrombectomy system (AngioDynamics, Inc, Latham, New York), a venovenous filtration apparatus used for aspiration of thrombi and/or vegetations, which could be used with clinical benefit in patients with right atrial thrombi; however, this system is used only in a small number of centers and there is limited literature about its efficacy (12).

## Data Availability Statement

The raw data supporting the conclusions of this article will be made available by the authors, without undue reservation.

## Ethics Statement

Written informed consent was obtained from the individual for the publication of any potentially identifiable images or data included in this article.

## Author Contributions

MB investigated the patient, collected and interpreted the data, and participated in manuscript preparation. CA collected and interpreted the data and participated in manuscript preparation. PA responsible for critical revising for intellectual content. RC interpreted the data and participated in manuscript preparation. CV investigated the patient, interpreted the data, and participated in manuscript preparation. All the authors critically revised the manuscript and approved the final version.

## Conflict of Interest

The authors declare that the research was conducted in the absence of any commercial or financial relationships that could be construed as a potential conflict of interest.

## Publisher's Note

All claims expressed in this article are solely those of the authors and do not necessarily represent those of their affiliated organizations, or those of the publisher, the editors and the reviewers. Any product that may be evaluated in this article, or claim that may be made by its manufacturer, is not guaranteed or endorsed by the publisher.
